# Adaptability of AI for safety evaluation in regulatory science: A case study of drug-induced liver injury

**DOI:** 10.3389/frai.2022.1034631

**Published:** 2022-11-08

**Authors:** Skylar Connor, Ting Li, Ruth Roberts, Shraddha Thakkar, Zhichao Liu, Weida Tong

**Affiliations:** ^1^National Center for Toxicological Research, US Food and Drug Administration, Jefferson, AR, United States; ^2^ApconiX Ltd., Macclesfield, United Kingdom; ^3^Department of Biosciences, University of Birmingham, Birmingham, United Kingdom; ^4^Center for Drug Evaluation and Research, US Food and Drug Administration, Silver Spring, MD, United States

**Keywords:** adaptability, AI, deep learning, drug-induced liver injury (DILI), drug safety, risk assessment, regulatory science

## Abstract

Artificial intelligence (AI) has played a crucial role in advancing biomedical sciences but has yet to have the impact it merits in regulatory science. As the field advances, *in silico* and *in vitro* approaches have been evaluated as alternatives to animal studies, in a drive to identify and mitigate safety concerns earlier in the drug development process. Although many AI tools are available, their acceptance in regulatory decision-making for drug efficacy and safety evaluation is still a challenge. It is a common perception that an AI model improves with more data, but does reality reflect this perception in drug safety assessments? Importantly, a model aiming at regulatory application needs to take a broad range of model characteristics into consideration. Among them is adaptability, defined as the adaptive behavior of a model as it is retrained on unseen data. This is an important model characteristic which should be considered in regulatory applications. In this study, we set up a comprehensive study to assess adaptability in AI by mimicking the real-world scenario of the annual addition of new drugs to the market, using a model we previously developed known as DeepDILI for predicting drug-induced liver injury (DILI) with a novel Deep Learning method. We found that the target test set plays a major role in assessing the adaptive behavior of our model. Our findings also indicated that adding more drugs to the training set does not significantly affect the predictive performance of our adaptive model. We concluded that the proposed adaptability assessment framework has utility in the evaluation of the performance of a model over time.

## AI in regulatory sciences

The term Artificial Intelligence (AI) refers to the ability of a computer system to learn from past data to predict future outcomes. Machine Learning (ML), a subset of AI, refers to the study and use of computer algorithms that automatically improve in making predictions or decisions based on their experiences and interactions with the training data (Gupta et al., 2021). Deep Learning (DL), a subset of ML, mimics the cognitive behaviors associated with the approach the human brain would take in learning and problem-solving of data-intensive problems (Gupta et al., 2021). Although AI has gained momentum in recent advancements within the biomedical field, especially in areas like drug safety evaluation and assessment, from a regulatory science perspective AI has yet to have the impact it merits.

Regulatory science is the science of developing new tools, standards, and approaches to assess the safety, efficacy, quality, and performance of FDA-regulated products (FDA, 2021). The main role of regulatory science is to certify the safety, proper labeling, and efficacy of food, drug and cosmetic items, like mandating food standards for packaging and quality, and regulating cosmetic products and medical devices (Patel and Miller, 2012). Despite being a critical component in the continued evolution of our approaches to certifying the safety and quality of food and medical products, regulatory science research has yet to have the impact it merits (Hamburg, 2011).

As the field of regulatory science advances, *in silico* and *in vitro* approaches have been extensively evaluated as alternatives to some animal studies, in a drive to identify and mitigate safety concerns earlier in the drug development process (Hamburg, 2011). AI and DL tools have begun to play a crucial role in the advancement of computer-aided drug discovery, design, and development (Gupta et al., 2021), specifically for the study of drug safety and efficacy. DL is arguably the most advanced ML approach that frequently outperforms conventional ML approaches (Slikker et al., 2012; Gupta et al., 2021; Anklam et al., 2022). DL usually consists of multiple layers of neural networks which can be constructed and connected in diverse ways, giving rise to a broad range of methodologies. As a result, DL has become the first-choice algorithm in regulatory science research due to its diversity and superior performance.

## Regulatory frameworks and the initiatives benefiting from AI

As interest in the use of AI within scientific and clinical research has grown, the global government agencies such as the European Medicines Agency, the European Food Safety Agency, the Unites States National Institute of Standards and Technology (NIST), the US Food and Drug Administration (FDA), and the United States Congress have worked to strengthen the guidance on how to safely implement the use of AI as software tools and medical devices. In 2021, the US House of Representatives introduced the FDA Modernization Act, H.R. 2565 (Text-H.R.2565-117th Congress (2021–2022), 2021) and S.2952 (Text-S.2952-117th Congress (2021–2022), 2021), intended to reform the drug approval process and drive the use of non-animal testing methods. In June 2022, the FDA Modernization Act as was passed as an additional provision, Section 701 (Text-H.R.7667-117th Congress (2021–2022), 2022), in a larger legislative package of FDA-related reforms known as the Food and Drug Amendments of 2022, H.R. 7667 (Text-H.R.7667-117th Congress (2021–2022), 2022). NIST has released several whitepapers providing guidance on how to properly implement AI in regulatory sciences like the 116^th^ Congress AI in Government Act of 2020 (Text-H.R.2575-116th Congress (2019–2020), 2020) and the 117^th^ Congress GOOD AI Act of 2021 (Text-S.3035-117th Congress (2021–2022), 2022).

The FDA has made major strides in guiding developmental and more recently computational opportunities within regulatory science through programs like the Drug Development Tool Qualification Programs (U. S. Food and Drug Administration, 2021a) and the FDA's Predictive Toxicology Roadmap (U. S. Food and Drug Administration, 2017), as well as many initiatives at the Center for Drug Evaluation and Research (CEDR) and for the first time an AI/ML specific Action Plan named “Artificial Intelligence/Machine Learning (AI/ML)-Based Software as a Medical Device (SaMD) Action Plan” has been instituted by the Center for Devices and Radiological Health (CDRH) (U. S. Food and Drug Administration, 2019b, 2021b).

In 2016 the FDA passed the Cures Act which defined a three-stage qualification process that allowed the use of qualified Drug Development Tools (DDTs) across drug development programs (U. S. Food and Drug Administration, 2021a). DDTs are methods, materials, or measures that have the potential to facilitate drug development. There is a total of four DDT Qualification Programs (U. S. Food and Drug Administration, 2021a). A qualified DDT has been determined to have a trusted specific interpretation and application within drug development and regulatory review for the qualified context of use. Once qualified, DDTs are made publicly available and can generally be included in Investigational New Drug (IND), New Drug Application (NDA), or Biologics License Application (BLA) submissions without requiring the FDA to reconsider or reconfirm its suitability (U. S. Food and Drug Administration, 2017, 2021a,c). The four programs, Animal Model, Biomarker, Clinical Outcome Assessment (COA), and the newest addition the Innovative Science and Technology Approaches for New Drugs (ISTAND) Pilot Program, rely on a context of use statement. The Context of use statement is one of the most important parts of the qualification process. The context of use should describe all elements that characterize the manner and purpose of use for the DDT being submitted (U. S. Food and Drug Administration, 2021a). Once qualified the context of use will define the boundaries that justify to others where they can use the qualified DDT. The ISTAND Pilot Program (U. S. Food and Drug Administration, 2021c) was developed to expand the current types of DDTs by encouraging the development and acceptance of DDTs that are outside of the scope of existing programs but are still novel approaches to drug development and acceptable for regulatory use. Once a new model is considered qualified by the FDA for a specific context of use, industry and other stakeholders may use it for the qualified purpose during product development without the need for FDA reviewers to re-review the underlying supporting data (U. S. Food and Drug Administration, 2017, 2021a,c).

In December of 2017 the FDA's Toxicology Working Group published the FDA's Predictive Toxicology Roadmap (U. S. Food and Drug Administration, 2017), a six-part framework outlining Agency priorities and engagement in predictive toxicology, and identifying current toxicology issues related to FDA-regulated products. The roadmap describes the FDA's current thoughts on practical ways to incorporate the development and evaluation of emerging toxicological methods and innovative technologies into the FDA regulatory review process. The six-part framework moves to enhance FDA engagement in the science of toxicology through the organization of a senior-level Toxicology Working Group that will help identify areas where research is needed, assist with efforts to reduce duplication and increase collaboration inside and outside the FDA through the encouragement of frequent communication and fostering collaborations across sectors and disciplines both nationally and internationally (U. S. Food and Drug Administration, 2021a).

## Adaptability of AI in regulatory science

Although there are several interpretations of adaptability and adaptive AI in the field, within this article we define adaptability as the study of the adaptive behavior of a model as it is retrained on unseen data. An adaptive model is a model that has the ability to continuously learn and change as it is used, meaning as time goes on the same question will not yield the same results as the model learns to better address the problem. A locked model is trained, developed, and tested to produce the best version of the model and once the model is launched for public or private use it should produce the same results every time the same input is used.

The AI/ML specific action plan was a response to a discussion paper published by the FDA in April of 2019 with a request for stakeholder feedback on the potential approach to the premarket review of AI and ML driven software modifications for Software used as a Medical Device (SaMD) (U. S. Food and Drug Administration, 2019b, 2021b). SaMD (Health et al., 2018) is “software intended to be used for one or more medical purposes that perform these purposes without being part of a hardware medical device” as defined by the International Medical Device Regulators Forum (IMDRF) (U. S. Food and Drug Administration, 2019a). As stated in the proposed plan, the FDA has cleared or approved several AI/ML-based SaMDs, but to date, SaMDs have typically only included algorithms that are “locked” prior to the systems or software's launch to market. Any proposed algorithm changes to a “locked” algorithm will likely require an FDA premarket review, especially if those changes are beyond the original approved authorization (U. S. Food and Drug Administration, 2019b). However, some algorithms have the capability and need to adapt over time through continuous learning from real-world experience after distribution.

The advantage and drawback, depending on the circumstance, of a “locked” algorithm is the fact it will not continually adapt or learn from its post market use, this feature is important in some instances but occasionally an adaptive algorithm is needed. The newly released AI/ML-Based SaMD Action Plan outlines five actions that the FDA intends to take to advance the use of AI/ML based software within regulatory science. The first of which is tailored toward the further development of adaptive AI and ML algorithms within the regulatory framework through the “issuance of Draft Guidance on the Predetermined Change Control Plan” which includes SaMD Pre-Specifications (SPS), where manufacturers describe “what” aspects they intend or anticipate modifying through continuously learning, and Algorithm Change Protocol (ACP) which explains “how” the algorithm will learn and change while remaining safe and effective (U. S. Food and Drug Administration, 2021b). The four other actions include encouraging the development of good ML practices, fostering a patient-centered approach through incorporating transparency to users, supporting regulatory science efforts to evaluate and improve ML algorithms; and working with stakeholders who are piloting the Real-World Performance (RWP) process for AI/ML-based SaMD.

Programs like ISTAND and the AI/ML-based SaMD Action Plan help lay the foundation for methodologies and tools to advance the use of computation within regulatory science. To test the assumption that drug safety models improve as more data is added to the training set, we set up a comprehensive study to mimic the real-world scenario of annually adding novel drugs to the market, using a model we previously developed for assessing drug-induced liver injury (DILI), known as DeepDILI (Li et al., 2021). In using this approach, we addressed two important questions: First, did the model's performance improve or decline as more data was added? Second, did the context of use change as the model adapted? Our evaluation followed the real-world scenario where a model was developed based on the drugs approved in the early years (before 1997) and assessed with the drugs approved thereafter (after 1997).

## DeepDILI: A deep learning model to evaluate drug-induced liver injury in humans

Evaluating DILI has been a persistent challenge for the past 60 years and continues to be the leading cause of toxicity failures in pharmaceutical development (PoPPer et al., 1965; Zimmerman, 1999; Van Norman, 2019). In our previous study, we developed an AI drug safety model, known as DeepDILI (Li et al., 2021), a deep learning-powered prediction model designed to identify drugs with DILI potential in humans solely based on chemical structure information. DeepDILI was created by combining model-level representation generated from five conventional ML algorithms [k-nearest neighbor (kNN), logistic regression (LR), support vector machine (SVM), random forest (RF), and extreme gradient boosting (XGBoost)] with a deep learning framework using Mold2 (Hong et al., 2008) chemical descriptors. With DeepDILI, we aimed to evaluate whether the DILI potential of newly approved drugs could be predicted by accumulating knowledge from previously approved drugs. For that reason, the DeepDILI model was trained with 753 drugs released to the market prior to 1997 and evaluated on the 249 drugs approved in 1997 and thereafter. Upon evaluation the model yielded an accuracy of 68.7%. In addition, DeepDILI was compared with a published DL DILI prediction model using three external validation sets, resulting in the DeepDILI model achieving better results with two data sets and comparable result with one.

## Adaptability of DeepDILI: An assessment based on a real-world scenario

**Figure 1:**
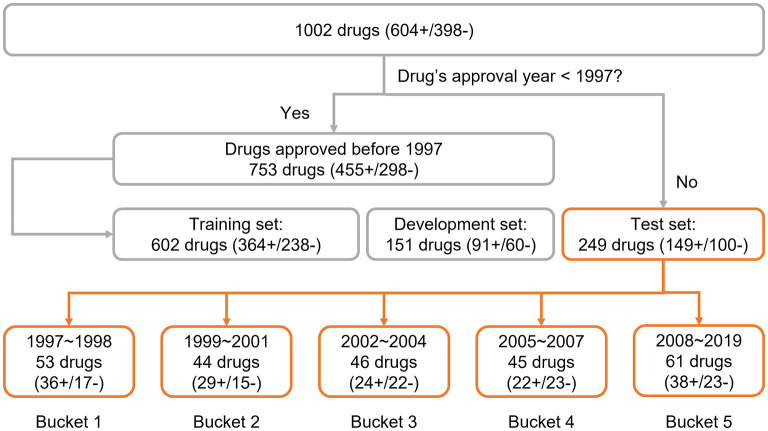
Data preparation: the data set was adopted from the previous DeepDILI study. The DeepDILI test set was split into five buckets based on the information of drugs' approval year. DILI positive and negative was labeled as “+”and “–”.

To explore the adaptability of an AI solution for drug risk, we implemented a time-split based adaptability framework using our DeepDILI prediction model (Li et al., 2021). We utilized our DILI Severity and Toxicity (DILIst) dataset, which is currently the largest binary human DILI classification data set (Thakkar et al., 2020). The 1,002 drugs from DILIst were first split based on the drugs' approval year; 753 drugs with an approval year before 1997 were used for model development and 249 drugs with an approval year after 1997 were used for testing. To implement a time-split adaptability framework analysis, the 249 drugs (with an approval year of 1997–2019) were split into five chronological groups or buckets of relatively the same size (Figure 1). Drugs approved from 1997 to 1998 were put into bucket 1, 1999 to 2001 in bucket 2, 2002 to 2004 in bucket 3, 2005 to 2007 in bucket 4, and 2008 to 2019 into bucket 5, with 53 (36+/17–), 44(29+/15–), 46(24+/22–), 45 (23+/22–), and 61 (38+/23–) drugs, respectively in each bucket (Figure 1). DILI positive and negative are labeled as “+”and “–”, respectively.

**Figure 2:**
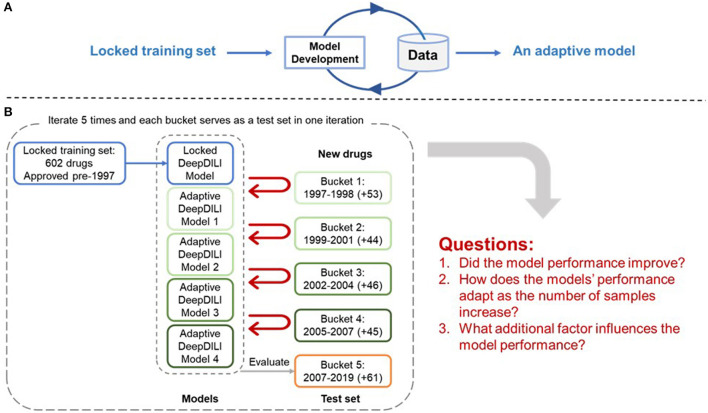
Adaptability Assessment Framework. **(A)** General framework of the adaptive model development, where the DeepDILI model adapts to new data by incorporating more data in the initial training set; **(B)** One iteration of the adaptability assessment process. In this iteration, bucket 5 was used as the test set, and the other four buckets served as the new drugs, that were chronologically and incrementally added to the initial training set. The process iterates five times as each bucket served as a test set.

The adaptability of DeepDILI was assessed by adding drugs from each of the previously mentioned buckets by year into the training set to develop adaptive DeepDILI models (Figure 2A). The new training set was used to develop a new and evolved DeepDILI model. More in depth details about the model development can be found in our previous DeepDILI work (Li et al., 2021). To mimic the real-world scenario of annually adding novel drugs to the market, we increased the number of new drugs by stepwise and chronologically adding each bucket of drugs. Through this method, there was at most four buckets of drugs added to the initial locked training set (i.e., the 753 drugs approved before 1997) and one bucket used for evaluating the performance of the adaptive models. For example, if bucket 5 containing drugs approved from 2008 to 2019 was used as the test set, the adaptative models were developed as follows (Figure 2B). The first adaptative model was developed with the locked training set (602 drugs approved before 1997) in addition to the new drugs from bucket 1 (53 drugs approved in 1997 and 1998) and evaluated with bucket 5 (61 drugs approved in 2008 to 2019). The second adaptive model was developed with the locked training set in addition to the new drugs from bucket 1 and bucket 2 (44 drugs approved in 1999 to 2001) and evaluated with bucket 5. The third adaptive model was developed with the locked training set in addition to the new drugs from buckets 1 through 3 (46 drugs approved in 2002 to 2004) and evaluated with bucket 5. The fourth adaptive model was developed with the locked training set in addition to the new drugs from buckets 1 through 4 (45 drugs approved in 2005 to 2007) and evaluated with bucket 5. Additionally, the performance of the four adaptive models were compared with that of the initial DeepDILI model with the test bucket, which in this case is bucket 5. This process was reiterated five times. Each time a different bucket served as the new test set and all remaining buckets were chronologically added to the training set as described above. The data and code are available through https://github.com/TingLi2016/Adaptability.

To assess the adaptative nature of DeepDILI, seven performance metrics were compared between the locked and adaptive DeepDILI models. We calculated seven performance metrics to evaluate the performance of the model: the area under the receiver operating characteristic curve (AUC), accuracy, sensitivity, specificity, F1, Matthew's correlation coefficient (MCC), and balanced accuracy (BA), were calculated using the following formulas:

*True Positive (TP), True Negative (TN), False Positive (FP), and False Negative (FN)*


(1) $$\begin{array}{l} accuracy=\frac{TP+TN}{TP+TN+FN+FP} \end{array}$$

(2) $$\begin{array}{l} sensitivity=\frac{TP}{TP+FN} \end{array}$$

(3) $$\begin{array}{l} specificity=\frac{TN}{TN+FP} \end{array}$$

(4) $$\begin{array}{l} F1=\frac{2TP}{2TP+FP+FN} \end{array}$$

(5) $$\begin{array}{l} MCC=\frac{TP^{*}TN-\;FP^{*}FN}{\sqrt{{\left(TP+FP\right)}^{*}{\left(TP+FN\right)}^{*}{\left(TN+FP\right)}^{*}\left(TN+FN\right)}}\\ \;BA=\frac{sensitivity+specificity}{2} \end{array}$$

MCC ranges from −1 to 1, with extreme values −1 and 1 representing perfect misclassification and perfect classification, respectively. All the other six metrics range from 0 to 1; a score of 1 indicates the model makes correct decision on every test case. Thus, the higher value the better. Although we evaluated seven metrics for the locked and adaptative DeepDILI models, it was common to find that one model had better performance in some metrics but may be inferior to other metrics during the model comparison. Therefore, we selected MCC as the main metric, which has proven to have advantages in the binary classifications for an unbalanced data set (Chicco and Jurman, 2020; Chicco et al., 2021).

### Key questions in adaptability assessment for the DeepDILI model

#### Has the model performance improved?

**Figure 3:**
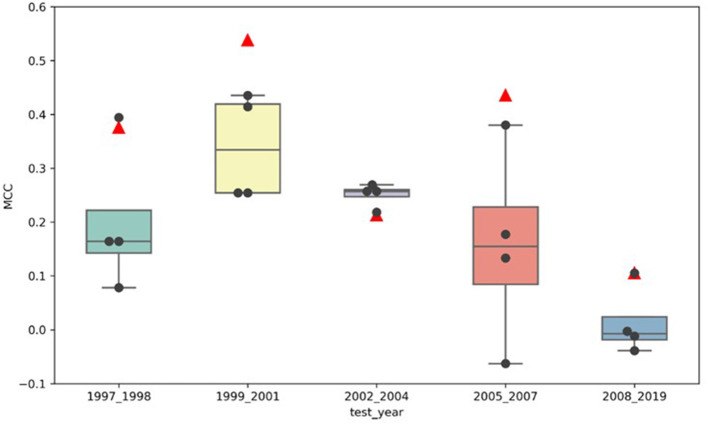
MCC distribution of the locked DeepDILI and adaptive DeepDILI models: the red triangle is the MCC of locked DeepDILI and the black dots represent the MCCs of the adaptive DeepDILI models for every test bucket. For example, 1997_1998 means that the tested drugs were approved in 1997 and 1998.

Figure 3 illustrates the comparison of the MCCs for the adaptive models (marked by the black dots) to the MCCs of the locked model (marked by the red triangles) for all five test sets, buckets 1–5. The locked DeepDILI model achieved the highest MCC of 0.538 and 0.436 in comparison to the adaptive models in the same test sets for bucket 2 (1999 to 2001) and bucket 4 (2005 to 2007), a comparable MCC of 0.376 and 0.106 in comparison to the adaptive models in the same test sets for bucket 1 (1997 to 1998) and bucket 5 (2008 to 2019), and the lowest MCC of 0.213 in comparison to the adaptive models in the same test sets for bucket 3 (2002 to 2004). Thus, we found that bucket 3 (2002 to 2004) was the only bucket in which the adaptive models MCC improved, as more drugs were added, in comparison to the locked DeepDILI model. The same trend was observed for the accuracy and F1, but a slight variance was found in the AUC, BA, sensitivity and specificity. Detailed information for these seven performance metrics can be found in Supplementary Table 1.

#### How does the performance of the model adapt as the number of drugs increases?

**Figure 4:**
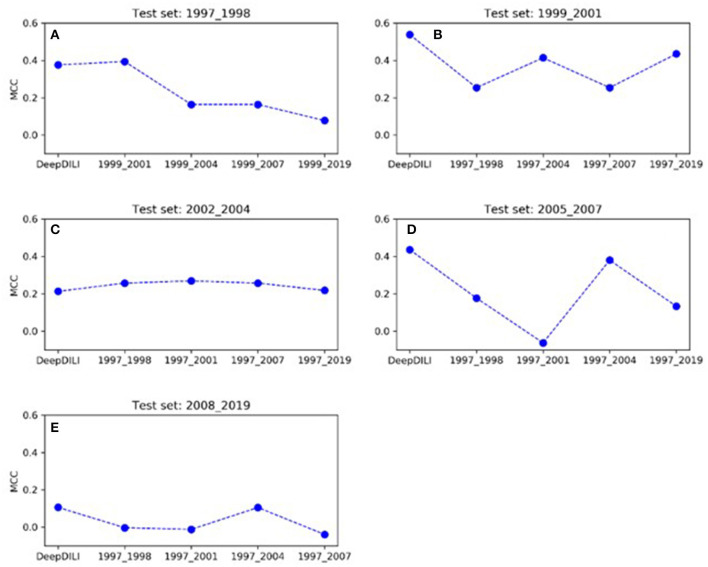
The trend of MCC among the locked DeepDILI and adaptive DeepDILI models within each buckets test set: for example, **(A)** showed the MCC trend of the locked DeepDILI model (labeled DeepDILI) and four adaptive DeepDILI models (labeled by the added drugs' approval year) on the test set with the drugs approved in 1997- and 1998. The following 4 sub-figures **(B–E)** follow this exact trend with their corresponding years.

To investigate whether the model performance was positively associated with the increasing number of drugs in the training set, we assessed the MCCs of the locked DeepDILI model (labeled as DeepDILI) and individual adaptive DeepDILI model for each test set (Figure 4). The locked DeepDILI model, which has the smallest number of drugs in the training set as compared to the adaptative DeepDILI models, was used as a baseline. In Figure 4A, the MCCs of the adaptive DeepDILI models for the test set of bucket 1 (1997 to 1998) decreased as more drugs were added to the training set. In Figures 4B,D, the MCCs of the adaptive models for the test sets of buckets 2 (1999 to 2001) and 4 (2005 to 2007) presented as a wave shape as more drugs were added to the training set. In Figures 4C,E, the MCCs of the adaptive models for the test sets of buckets 3 (2002 to 2004) and 5 (2008 to 2019) exhibited a relatively flat trend as more drugs were added to the training set, indicating that as more drugs were used in the training, the performance of the adaptive models did not improve. Thus, there is no positive relationship between the model performance and the number of drugs in the training set. In addition, no general pattern was found in the adaptive models performance as we increased the number of drugs in the training set.

#### What additional factors influence the models' performance?

As the performance of the models adapted to the addition of new drugs, we observed the average MCC varied from one test set to another (Supplementary Table 1). The test set of bucket 2 (1999 to 2001) achieved the highest average MCC of 0.379, while bucket 5 (2008 to 2019) yielded the lowest average MCC of 0.031. The test sets of buckets 1 (1997 to 1998), 3 (2002 to 2004), and 4 (2005 to 2007) yielded similar average MCCs of 0.235, 0.243, and 0.213, respectively. This indicates that different test sets presented various levels of challenges for DILI prediction, showing that the properties of the test set data are a key factor in the model's performance.

## Discussion

Although AI is promising, there is still work to do; a comprehensive assessment of the adaptive behavior and context-of-use of AI models for regulatory application is required. As two important aspects of regulatory significance, especially for the application of AI, the applicability domain and context of use play a significant role in enhancing AI solutions for risk assessments within the regulatory arena. On every occasion, the context of use should clearly convey to users where the model is best utilized as well as whether the model is intended to complement or replace current technologies (Anklam et al., 2022), while the applicability domain outlines how the model is used through defining best practices (Anklam et al., 2022).

When it comes to using adaptive models and assessing their adaptive behavior there are a number of strategies and approaches being used across the field of AI (Groce et al., 2002; Yang et al., 2005; Xiao et al., 2016; López and Tucker, 2018). Currently, a random split cross-validation model is considered the ML standard for model building and evaluation (Morita et al., 2022). Random split cross-validation is often found to be overoptimistic in comparison to real-world situations, while a time-split approach is considered suitable for real-world prediction (Morita et al., 2022). In this study, we proposed a time-split adaptability framework approach to exploring the adaptive behavior of an AI-based solution for drug toxicity and risk assessments within regulatory science. In using the time-split approach, we were able to discuss two important questions: (1) Did the models performance improve or decline as more data was added? And (2) Did the context of use change as the model adapted?

Through the real-world scenario of annually adding new drugs to the market to retrain our model, we found that the target test set plays a major role in the adaptive behavior of our model. Our findings suggest that regardless of the individual model performance, the average MCC was found to vary from one test set to another. This indicates that different test sets possess different levels of challenge for prediction, demonstrating that the target test set appears to be the most important factor in performance. The context of use for our DeepDILI model was the same for the locked and adaptive models. DeepDILI aims to flag the human DILI potential of DILI positive drugs using the chemical structure that have a molecular weight lower than 1,000 g/mol. Since these criteria were used to screen the drugs for the initial model that our adaptive framework was remodeled from our context of use did not change as the model adapted to “new” data. Although a time-split approach is seen to be better for real-world prediction, a major caveat of this approach are the limitations with respect to the amount of usable or available data for model training, development, and testing. In future studies, it would be beneficial to assess the application of our adaptive framework to other types of predictive models to determine their adaptive behavior. Since drug induced organ injury is a leading cause of drug withdrawals, it would be beneficial to see how our locked and adaptive model frameworks perform when used on other organ systems.

Our results indicated that adding more drugs to the training set did not substantially contribute to the performance of the adaptive DeepDILI model. Overall, based on these findings we conclude that the proposed adaptability assessment framework has utility in the evaluation of a model's adaptive performance over time, which would greatly support the advancement of AI-based models in regulatory science. Using comprehensive assessments to evaluate the adaptive behavior and context-of-use of AI based safety evaluation and risk assessment models, whether locked or adaptive, can have a positive impact on decision making within regulatory science. Currently, reviewers utilize animal pharmacology and toxicology data, manufacturing information, clinical protocols and any past knowledge of the compound to assess the safety of a new drug. The development and parallel use of alternative approaches to identify and signal different safety concerns earlier in the review process are essential to the future of regulatory science.

## Author contributions

WT devised the study. SC and TL wrote the manuscript and performed data analysis. WT, ZL, RR, and ST revised the manuscript. All authors read and approved the final manuscript.

## Conflict of interest

Author RR is co-founder and co-director of ApconiX, an integrated toxicology and ion channel company that provides expert advice on non-clinical aspects of drug discovery and drug development to academia, industry, and not-for-profit organizations. The remaining authors declare that the research was conducted in the absence of any commercial or financial relationships that could be construed as a potential conflict of interest.

## Publisher's note

All claims expressed in this article are solely those of the authors and do not necessarily represent those of their affiliated organizations, or those of the publisher, the editors and the reviewers. Any product that may be evaluated in this article, or claim that may be made by its manufacturer, is not guaranteed or endorsed by the publisher.

## Author disclaimer

This manuscript reflects the views of the authors and does not necessarily reflect those of the Food and Drug Administration. Any mention of commercial products is for clarification only and is not intended as approval, endorsement, or recommendation.
