# Supplementary material for: Adaptability of AI for safety evaluation in regulatory science: A case study of drug-induced liver injury
Source: Front Artif Intell. 2022 Nov 8;5:1034631. doi: 10.3389/frai.2022.1034631 (PMC9679417; doi:10.3389/frai.2022.1034631)
Supplement: Supplementary file 1 [file Table_1.DOCX]

Supplementary Material

**Supplementary Table 1:** Performance metrics for the locked DeepDILI and the adaptive DeepDILI models

| **Test Set** | **Models** | **MCC** | **Accuracy** | **AUC** | **F1** | **Balanced** | **Sensitivity** | **Specificity** |
| --- | --- | --- | --- | --- | --- | --- | --- | --- |
|  |  |  |  |  |  | **Accuracy** |  |  |
| **Bucket 1**  **1997_1998** | Locked DeepDILI | 0.376 | 0.736 | 0.757 | 0.811 | 0.681 | 0.833 | 0.529 |
|  | 1999_2001 | 0.394 | 0.736 | 0.729 | 0.806 | 0.697 | 0.806 | 0.588 |
|  | 1999_2004 | 0.164 | 0.642 | 0.598 | 0.740 | 0.581 | 0.750 | 0.412 |
|  | 1999_2007 | 0.164 | 0.642 | 0.642 | 0.740 | 0.581 | 0.750 | 0.412 |
|  | 1999_2019 | 0.078 | 0.623 | 0.637 | 0.737 | 0.536 | 0.778 | 0.294 |
| **Bucket2**  **1999_2001** | Locked DeepDILI | 0.538 | 0.795 | 0.784 | 0.847 | 0.764 | 0.862 | 0.667 |
|  | 1997_1998 | 0.254 | 0.659 | 0.664 | 0.737 | 0.629 | 0.724 | 0.533 |
|  | 1997_2004 | 0.414 | 0.727 | 0.669 | 0.786 | 0.713 | 0.759 | 0.667 |
|  | 1997_2007 | 0.254 | 0.659 | 0.657 | 0.737 | 0.629 | 0.724 | 0.533 |
|  | 1997_2019 | 0.435 | 0.750 | 0.752 | 0.814 | 0.714 | 0.828 | 0.600 |
| **Bucket3**  **2002_2004** | Locked DeepDILI | 0.213 | 0.609 | 0.621 | 0.654 | 0.604 | 0.708 | 0.500 |
|  | 1997_1998 | 0.257 | 0.630 | 0.614 | 0.667 | 0.627 | 0.708 | 0.545 |
|  | 1997_2001 | 0.269 | 0.630 | 0.653 | 0.702 | 0.621 | 0.833 | 0.409 |
|  | 1997_2007 | 0.257 | 0.630 | 0.576 | 0.667 | 0.627 | 0.708 | 0.545 |
|  | 1997_2019 | 0.218 | 0.609 | 0.597 | 0.679 | 0.600 | 0.792 | 0.409 |
| **Bucket4**  **2005_2007** | Locked DeepDILI | 0.436 | 0.711 | 0.648 | 0.735 | 0.713 | 0.818 | 0.609 |
|  | 1997_1998 | 0.177 | 0.578 | 0.559 | 0.642 | 0.582 | 0.773 | 0.391 |
|  | 1997_2001 | -0.063 | 0.467 | 0.494 | 0.538 | 0.470 | 0.636 | 0.304 |
|  | 1997_2004 | 0.38 | 0.689 | 0.767 | 0.696 | 0.690 | 0.727 | 0.652 |
|  | 1997_2019 | 0.133 | 0.556 | 0.648 | 0.630 | 0.560 | 0.773 | 0.348 |
| **Bucket5**  **2008_2019** | Locked DeepDILI | 0.106 | 0.607 | 0.538 | 0.714 | 0.547 | 0.789 | 0.304 |
|  | 1997_1998 | -0.003 | 0.557 | 0.525 | 0.675 | 0.499 | 0.737 | 0.261 |
|  | 1997_2001 | -0.012 | 0.541 | 0.49 | 0.650 | 0.494 | 0.684 | 0.304 |
|  | 1997_2004 | 0.105 | 0.590 | 0.562 | 0.684 | 0.551 | 0.711 | 0.391 |
|  | 1997_2007 | -0.039 | 0.525 | 0.501 | 0.633 | 0.481 | 0.658 | 0.304 |
